# GLP-1/GLP-1R axis: from metabolism (obesity and T2DM) to immunity

**DOI:** 10.1098/rsob.240303

**Published:** 2025-07-02

**Authors:** Vijay Kumar

**Affiliations:** ^1^ Department of Surgery, Morehouse School of Medicine, Atlanta, GA, USA

**Keywords:** GLP-1/GLP-1R axis, L cells, immunity, hypothalamus, macrophages, intraepithelial lymphocytes

## Abstract

The discovery of glucagon-like peptide-1 (GLP-1) has revolutionized metabolism research in the context of obesity and type 2 diabetes mellitus (T2DM). For example, worldwide, more than 537 million adults are affected with T2DM, and more than 30.3 million people in the USA alone are suffering from T2DM. Obesity is one independent risk factor for T2DM; therefore, targeting obesity may lower the T2DM development risk. Hence, pharmaceutical companies have developed different GLP-1R agonists (GLP-1RAs) to target obesity and T2DM, which comprised multibillion-dollar businesses. However, metabolism and immune response are well-correlated processes that affect each other. For example, recent advances in metabolic processes governing the immune response have led to the evolution of *immunometabolism*, which can be divided into cellular, tissue and systemic immunometabolism. The current open-question article is intended to explore the impact of the GLP-1/GLP-1R axis on the immune response governed by the functioning of various immune cells and their interaction with the nervous system and microbiota axis that further depends on the gender and circadian clock of the host. Along with food/sugar ingestion, several other factors controlling the GLP-1 secretion and its immunomodulatory functions have been discussed to highlight the importance of the GLP-1/GLP-1R axis in immunoregulation. Therefore, understanding the GLP-1/GLP-1R axis/interaction at the immunological level will help to understand the adverse events associated with GLP-1RAs and their use as an immunomodulatory agent in acute and chronic inflammatory conditions depending on the gender and metabolic status of the host.

**Figure:**
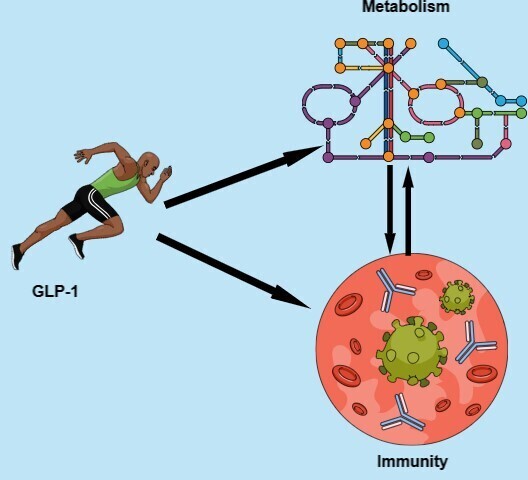

## 1. Introduction

The discovery of glucagon-like peptide-1 (GLP-1; a 30-amino-acid peptide hormone) has become one of the great discoveries of the twentieth century as its discoverers (Joel Hebener, Svetlana Mojsov and Lotte Bjerre Knudsen) and developers of GLP-1 receptor agonists (GLP-1RAs) to target obesity and type 2 diabetes mellitus (T2DM) have been awarded 2024 Lasker-DeBakey Clinical Medical Research Award [1,2]. The GLP-1 discovery and development of its agonists have revolutionized biomedical research focusing on metabolic disorders, such as obesity, T2DM, atherosclerosis and cardiovascular diseases (CVDs) and pharmaceutical industrial revenue. For example, at least six pharmaceutical companies, such as Eli Lilly (Zepbound mimics GLP-1 and glucose-dependent insulinotropic polypeptide got USFDA approval in 2023 as weight loss drug), Novo Nordisk (semaglutide sold as wegovy got USFDA approval as a weight loss drug in 2021), Teva Pharmaceuticals (launched Victozoa a generic GLP-1 agonist, which is similar to ozempic), Pfizer (GLP-1R agonist, danuglipron tromethamine is in phase II clinical trial) and Sciwind Biosciences (GLP-1R agonist, XW0003 or ecnoglutide is under phase III clinical trial), are in the race for GLP-1-mediated targeting of metabolic diseases.

**Figure 1.**
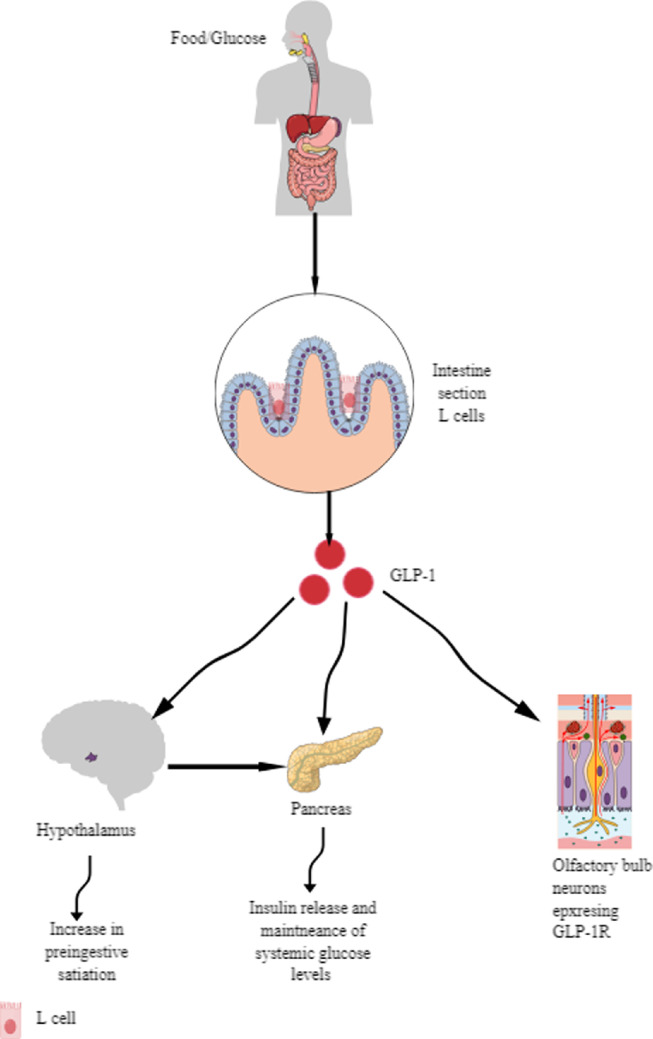
Schematic representation of GLP-1/GLP-1R axis in maintaining systemic glucose level. Ingestion of glucose or food enriched in sugar and lipids induces the GLP-1 release from intestinal L cells. In addition, hypothalamus and olfactory bulb neurons expressing GLP-1Rs also release GLP-1 and associated systemic glucose concentration control by stimulating insulin release from the pancreas. Details are mentioned in the text.

GLP-1 is secreted by intestinal epithelial endocrine cells (IEECs), called L cells of the ileum and colonic mucosae of the large intestine, in response to food/oral sugar or glucose intake (figure 1). In L cells, the proglucagon (the GLP-1 precursor) undergoes different enzymatic cleavage steps to generate glicentin, GLP-1 and GLP-2 [3]. GLP-1 is an intestinal glucagon to control systemic glucose levels (figure 1) [3,4]. The ileal L cells produce higher GLP-1 than the ascending and transverse colon, similar to the GLP-1 produced by the sigmoid and ascending colon [5]. Interestingly, GLP-1 secretion from ileal L cells decreases with increased body mass index (BMI), whereas colonic L cell-mediated GLP-1 production decreases with ageing. Furthermore, chronic TNF-α exposure to intestinal L cells decreases GLP-1 secretion and anti-TNF-α antibody; etanercept treatment reverses this effect in male mice with high fat diet (HFD)-induced obesity, which enhances the ageing process [6,7].

It is critical to note that truncated GLP-1 is a potent activator of glucose-induced insulin secretion, and full-length GLP-1 is inactive [8,9]. Thus, out of two GLP-1 isoforms: (i) GLP-1 with 37 amino acids (GLP-1(1-37)) and (ii) GLP-1 with 31 amino acids (GLP-1(7-37)) only GLP-1(1-37) is critical to control physiological insulin secretion. Furthermore, the GLP-1 secreting L cells or preproglucagon cells are present in the olfactory bulb (OB), and the GLP-1 receptor activation in the OB stimulates insulin release in response to sugar intake in normal and mice (male) with diet-induced obesity (figure 1) [10]. OB/GLP-1/GLP-1R axis-mediated insulin secretion involves sympathetic nervous system (SNS) inhibition, specifically sympathetic nerve activity to the pancreas. For example, inhibiting gamma amino butyric acid (GABA)A receptors with bicuculline in the hypothalamic paraventricular nucleus (PVN, central regulator of SNS) in mice having Western diet (WD) suppresses insulin release in response to OB/GLP-1/GLP-1R axis activation [10]. Thus, GLP-1 through gastrointestinal tract (GIT) and neuronal regulation controls insulin secretion to control metabolism, such as glucose metabolism and obesity (figure 1).

Interestingly, the immune system also plays a critical role in the pathogenesis of metabolic disorders, such as obesity, T2DM, atherosclerosis and CVDs or vice versa [11–14]. Moreover, women taking ozemic and wegoy (semaglutide) have reported an increase in unplanned pregnancies, which has also been reported in mice treated with GLP-1 receptor agonist (GLP-1RA) liraglutide [15,16]. Additionally, GLP-1RAs have improved natural pregnancy rate, menstrual cyclicity and hormonal indexes in women with polycystic ovary syndrome (PCOS) [17]. The immune system is critical in regulating male and female fertility [18–22]. Furthermore, male and female sex hormones (testosterone and estrogen) exhibit different metabolic effects depending on their concentrations in the two genders, which must be considered at clinical levels [23]. Therefore, exploring the impact of GLP-1/GLP-1R interaction or GLP-1 agonists on the human immune system would be interesting, which critically determines their wellbeing and resistance to diseases, including infections, cancers and other inflammatory diseases depending on gender and metabolic status. The current article explores the missing link of the GLP-1/GLP-1R axis in immunity and immune homeostasis.

## 2. Metabolism is a key to healthy immunity or immune response

Metabolism regulates the provision of nutrients to the body’s cellular system based on metabolic demand, which depends on their growth, proliferation and division status, determining their function. Nutritional status critically regulates immune cell metabolism and function [24]. For example, immune cells, such as macrophages, dendritic cells (DCs), T cells, B cells and NK cells at their steady or homeostatic state, do not require high energy at a frequent rate and therefore utilize oxidative phosphorylation (OXPHOS), mainly along with other metabolic pathways to meet their metabolic demand [25–29]. However, immune cells become hyperactive during infection or inflammatory conditions, and OXPHOS shifts to frequent energy-supplying glycolysis. The glycolysis provides only two adenosine triphosphate (ATP) molecules per cycle more frequently than OXPHOS to support their increased immunological functions and their survival, growth, division and proliferation. The metabolic process controlling immune cell function and phenotype is called *immunometabolism,* which can be *cellular*, *tissue* and *systemic immunometabolism* as described elsewhere [30–32]. Hence, pathogenic infections, sterile inflammatory diseases, including cancers, modify metabolism depending on the pathogen and cancer type and origin, including immunometabolism, to escape from the detrimental host immune response for their survival and spread (metastasis in cases of cancers) [33–39]. For example, fasting metabolism is protective in bacterial infections causing sepsis, and nutritional supplementation becomes detrimental during bacterial sepsis, which is opposite during viral infections, such as influenza and viral sepsis [40,41]. Hence, chronic alteration of metabolism may serve as a critical factor for an altered immune response governing immunity against infections, cancers, and other inflammatory disorders.

## 3. GLP-1-mediated immunoregulation through different (neuronal and metabolic) mechanisms

**Figure 2.**
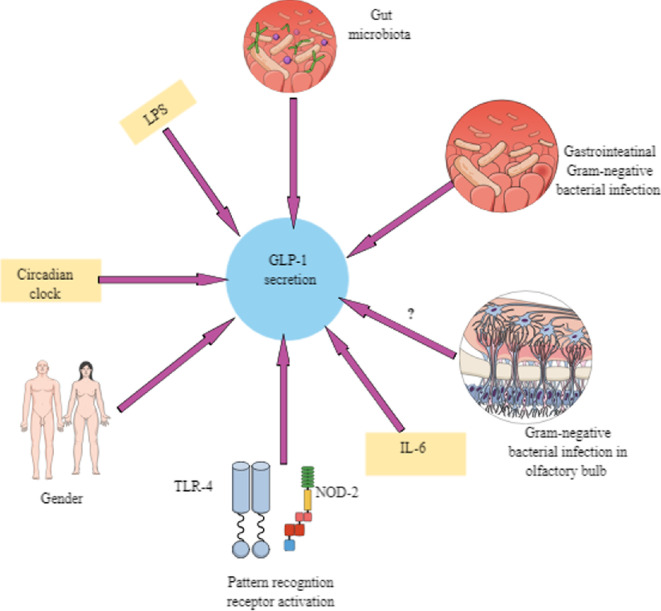
Factors other than dietary glucose/fat controlling GLP-1 release. In addition to dietary sugar and fat, GLP-1 release from intestinal L cells is influenced/controlled by several other factors, such as gut microbiota, systemic inflammation as seen during sepsis, endotoxaemia (LPS), gastrointestinal infection with Gram-negative bacteria, activation of different pattern recognition receptors, such as TLR4, NOD2 and CD14, gender, circadian clock alteration and systemic IL-6 level. As olfactory bulb neurons also release GLP-1 and express GLP-1Rs, it is interesting to see how infections in the olfactory bulb affect the GLP-1/GLP-1R axis and immune response. Details are mentioned in the text*.*

The numbers and activity of GLP-1-producing L cells are affected by several factors. For example, pharmacological inhibition of Notch or Ras homologue family member A (RhoA) signalling via Rho-associated coiled-coil-containing protein kinases 1 and 2 (ROCK1 and ROCK2) in mice and human intestine organoids increased the numbers of functional L cells releasing several folds of GLP-1 release [42]. Furthermore, in patients undergoing Roux-en-Y gastric bypass weight loss surgery restricting food intake and preventing nutrient absorption, L cell number and GLP-1 production increase [43]. Systemic inflammatory conditions, including endotoxemia associated with increased pro-inflammatory cytokines, such as IL-6, also increase GLP-1 production from L cells (figure 2) [44–46]. The increased systemic plasma level of GLP-1 in critically ill patients with sepsis and chronic kidney disease (CKD) admitted to the intensive care unit (ICU) correlates well with inflammation markers and disease severity [46,47]. The increased systemic GLP-1 level further independently correlates and predicts the mortality of critically ill and end-stage renal disease patients admitted in ICUs, serving as an independent predictor of patient survival and providing a superior prognostic measure than circulating C-reactive protein (CRP) as an indicator of systemic inflammation and systemic creatinine level as a marker of kidney disease [47]. The elevated GLP-1 in systemic inflammatory conditions, such as sepsis, might be another anti-inflammatory mechanism to compensate for exaggerated inflammation as decreasing glucose intake during bacterial infections leading to sepsis onset protects from lethality, including neuronal damage [40] and dietary glucose intake is one of the other macronutrients regulating gastric and neuronal GLP-1 release. For example, lipopolysaccharide (LPS)-induced endotoxaemia increases glucose uptake in the hypothalamus, where it can increase the GLP-1 production from hindbrain GLP-1 neurons that acts on GLP-1Rs expressed on lateral hypothalamus (LH) to induce anorexia as GLP-1 is an anorexigenic peptide [40,48,49]. Another study has indicated that the loss of GLP-1R in *Phox2b^+^* cells present in nodose ganglion (NG), midbrain, hindbrain and visceral sensory neurons impairs glucose homoeostasis, which can be altered during different stressful conditions, such as bacterial sepsis [50]. Whereas viral infection or polyinosinic:polycytidylic acid (polyI:C, a synthetic double stranded (ds) RNA) treatment induces glucose uptake in the brainstem, which does not have a direct effect on increasing neuronal GLP-1 production and inducing anorexia. Therefore, it is critical to understand the role of GLP-1 in bacterial and viral infections to target the GLP-1/GLP-1R axis for increasing the efficacy of available therapeutics, specifically for sepsis management.

Experimental studies have indicated that GLP-1RA (liraglutide) treatment in mice with endotoxemia increases survival and decreases inflammatory markers (monocyte chemoattractant protein-1 (MCP-1 or chemokine (C-C motif) ligand 2 or CCL2), TNF-α, inducible nitric oxide synthase or iNOS, intercellular adhesion molecule 1 or ICAM-1 and vascular cell adhesion molecule-1 or VCAM-1) in leukocytes and endothelial cells (ECs) and vascular dysfunction [51,52]. Furthermore, the protective action of liraglutide increases with dipeptidyl peptidase-4 (DPP-4, an endogenous GLP-1 degrading enzyme) inhibitor, linagliptin. The protective action of linagliptin is adenosine monophosphate (AMP)-activated protein kinase (AMPK)-dependent [51]. However, IL−6 is a significant pro-inflammatory marker for the GLP-1 release in inflammatory conditions independent of the inflammogen source, such as infections and non-infectious inflammatory conditions, including T2DM, CKD and surgery-induced trauma (figure 2) [53].

The hindbrain also produces GLP-1 and GLP-1Rs are distributed throughout the central energy-balance-regulating system comprising the hypothalamus, thalamus and hindbrain [54,55]. The neurons in the caudal portion of the nucleus of the solitary tract (NTS) are the primary endogenous source of GLP-1 in the brain and its GLP-1Rs. In contrast, neurons in the central portion of the NTS are catecholaminergic [55,56]. The central GLP-1R stimulation with GLP-1RA (exendin-4, a 39-amino-acid peptide isolated from the saliva of Gila monster, *Heloderma suspectum*) upregulates IL-6 and IL-1β production in the hypothalamus and hindbrain [57,58]. Furthermore, central injection of exendin-4 elevates hypothalamic and hindbrain interleukin-associated intracellular signals (phosphorylated signal transducer and activator of transcription-3 (pSTAT3) and suppressor of cytokine signalling-1 (SOCS1)). However, blocking CNS, IL-1 and IL-6 receptors (IL-1R and IL-6R) attenuates exendin-4-induced anorexia and weight loss. Furthermore, with global IL-1R gene knockout or central IL-6R knockdown, peripheral treatment with exendin-4 loses its anorexic and weight loss effect [57]. A recent study has further indicated the promotion of subcutaneous fat retention in humans upon basal IL-6 inhibition during fasting and postprandial states due to diminished fatty acid uptake and oxidation in skeletal muscles [59]. Therefore, the involvement of basal IL-6 level in GLP-1 release must be investigated as increased IL-6 under different pro-inflammatory conditions increases GLP-1 production to exert anorexic and immunomodulatory effects.

Microglia are potent immune cells in the brain to release IL-6 [60]; therefore, exploring GLP-1/GLP-1R axis-induced IL-6 and IL-1β release in the hypothalamus and hindbrain would be interesting. For example, basal hypothalamic IL-6 level and microglia function maintain hypothalamus homeostasis, which is critical to GLP-1/GLP-1R-mediated food intake control, and its disturbance may impact systemic infections, such as bacterial sepsis and other inflammatory conditions as seen in obesity [60–62]. However, chronic IL-6 loss in the LH induces weight gain due to hyperphagia in male rats but not in females [63]; therefore, the gender-dependent impact of LH IL-6 and GLP-1/GLP-1R axis in humans is critical to establish (figure 2). Furthermore, the hypothalamic IL-6-mediated ERK1/2 pathway in the ventromedial hypothalamus (VMH) via the α2-adrenergic pathway induces sustained AMPK and ACC phosphorylation and fatty acid oxidation (FAO) in murine and human skeletal muscles [64]. Thus, hypothalamic IL-6 through different mechanisms, including the GLP-1/GLP-1R axis, is critical to maintaining metabolism and, thus, inflammatory events.

A recent study has brought to light a significant contradiction. It suggests that specific targeting of the NTS GLP-1R neurons (dorsal vagal complex or DVC and vagal afferents or the nodose ganglion or NG) for weight loss could avoid adverse events, such as nausea and vomiting associated with GLP-1R agonists [65]. This finding challenges the prevailing belief in the major involvement of the hypothalamus in the suppression of food intake and the reduction of obesity in patients receiving GLP-1RAs to treat obesity and T2DM. Another recent murine study has indicated that hypothalamus GLP-1Rs are not critical to induce liraglutide, GLP-1AR agonist-associated reduced food intake and weight, instead GLP-1R-positive neurons of lateral septum, a brain region projecting keys to its feeding centres and controls gastric emptying following food consumption [66,67]. Thus, the role of the hypothalamic GLP-1/GLP-1R axis in maintaining metabolism through the endogenous GLP-1 is underscored, while the role of exogenous GLP-1RAs is brought into question.

However, these studies have not yet explored the potential impact of the immune response on reducing food intake and aversion behaviour in response to GLP-1RAs used in the study. For instance, peripheral pro-inflammatory immune response activates caudal NTS (cNTS, a major first stop for incoming information from the body to the brain carried by the vagus nerve) via the vagus nerve, and the inhibition of cNTS neurons increases pro-inflammatory response and decreases a concomitant anti-inflammatory immune response [68,69]. cNTS serves as a homoeostatic neural control of peripheral immune response, and therefore pharmacological targeting of cNTS neurons may impact the host’s peripheral immunity, which can be determinant to the antimicrobial and antitumor immunity. The involvement of the vagus nerve in sickness (immunological) and social and feeding behaviours has been discussed elsewhere [70,71]. Hence, the GLP-1/GLP-1R axis may serve as a missing link between neuronal regulation of immunity and the control of metabolic regulation of inflammation, opening up a new avenue for research and potential therapeutic interventions.

## 4. GLP-1/GLP-1R axis in direct cellular and humoral immunoregulation

Upon recognizing gastrointestinal infection, inflammation and altered gut microbiota, L cells release GLP-1. For example, *Akkermansia muciniphila* (a beneficial anaerobic Gram-negative bacterium comprising 1–4% of the total foecal microbiota) secretes P9, an 84 kDa protein, which interacts with ICAM-2 expressed by L cells stimulating the release of GLP-1, which is further increased by IL-6 that may be released due to toll-like receptor (TLR2 and 4) activation (figure 2) [72–78]. *A. muciniphila* increases IL-6 production in the ileum and colon, and the absence of IL-6 limits GLP-1 production by L cells. Thus, bacterial P9 protein and TLR4 activation stimulate the IL-6/GLP-1/GLP-1R axis to exert metabolic and immunomodulatory actions. IL-6 is critically needed under physiological conditions to maintain intestinal epithelial barrier, intestinal epithelial cell proliferation, intestinal stem cell niche and mucin production to maintain the gut homoeostasis, including its healing post-intestinal injury/inflammation [79–83]. Furthermore, LPS-producing bacteria may also stimulate L cells to produce GLP-1 via TLR4 activation in the inflamed and injured GIT prior to the systemic spread of infection and inflammation (figure 2) [84–86]. Interestingly, TLR4^−/−^ mice subjected to caecal-ligation and puncture (CLP)-induced sepsis also produce GLP-1 like wild-type (WT) mice [87]. Thus, activation of other TLRs might also be involved in GLP-1 production in the gut. Hence, early GLP-1 production during localized GIT inflammation attempts to contain the infection/inflammation locally by inducing local anti-inflammatory mechanisms.

IL-6 also significantly increases GLP-1 production from L cells in response to LPS-mediated TLR-4 activation without inducing glucose-dependent insulinotropic polypeptide (GIP) [84]. Even atropine-mediated muscarinic neural transmission blockage did not significantly lower GLP-1 production in GI L cells upon stimulation with LPS. The LPS-mediated L cell TLR4 activation increases cytosolic calcium (Ca^2+^) as seen in other immune cells, such as macrophages, astrocytes and endothelial cells (ECs), by activating different Ca^2+^ channels (transient receptor potential melastatin-like 7 (TRPM7) channel in macrophages, Orai1, a key component of calcium release-activated calcium channels or CRACs in astrocytes, and transient receptor potential canonical channel 6 or TRPC6 in ECs) [88–90].

Interestingly, LPS treatment also stimulates GLP-1 production in human L cells *in vivo*. Thus, in humans, GLP-1 is released as an anti-inflammatory molecule/cytokine to contain gastrointestinal inflammation at earlier stages. Furthermore, the GLP-1/GLP-1R axis controls high-fat diet (HFD)-induced altered microbiota-associated chronic inflammation, including hypothalamus inflammation, by maintaining enhanced leptin sensitivity along with maintaining colonocyte homoeostasis and metabolic energy status [91,92]. The GLP-1/GLP-1R axis circadian rhythmicity and gut microbiota are interdependent (figure 2) and depend on timing and diet components, which may affect the circadian rhythmicity of the immune response and metabolism, depending on the timing of GLP-1RAs administration in patients [93–99]. Thus, the altered GLP-1/GLP-1R axis has the potential to modulate immune response through different mechanisms, such as metabolic alterations, microbiota alteration, neuronal regulation, circadian rhythmicity and direct interaction with potent immune cells, as discussed below.

Further study has suggested that a specific set of gut microbiota in the ileum impair the GLP-1-mediated gut–brain axis controlling insulin secretion and gastric emptying, indicating that GLP-1RAs will not work in all T2DM patients and must be discontinued in those patients with altered microbiota and enteric neuron-mediated nitric oxide (NO^.^) release [100]. The Gram-negative bacterial infection of the GIT activating the TLR4 signalling pathway in male rats also induces GLP-1 secretion from L cells expressing TLR4. It increases colonic peristalsis by stimulating the calcitonin gene-related peptide (CGRP)-containing neurons [101]. However, sterile inflammatory conditions, such as obesity and other metabolic syndrome-associated ailments with altered microbiota may exhibit altered or a decreased GLP-1 production and a protective TLR4 signalling, which is seen during intact epithelial barrier integrity and gut microbiota.

For example, nucleotide-binding oligomerization domain 2 (NOD2, a cytosolic sensor of muramyl dipeptide (MDP), a component of the peptidoglycan (PGN) present in the bacterial cell wall), TLR4 and CD14 KO mice produce lower GLP-1 levels (figure 2) and exhibit altered gastric emptying in response to HFD, indicating gut microbiota and their recognition by intestinal PRRs critical to induce the IL-6 release are critical factors for GLP-1 production [100,102–104]. HFD in mice suppresses MDP-induced GLP-1 secretion, and L cells isolated from hyperglycaemic mice have reduced GLP-1 and NOD2 expression [105]. For example, TLR4 and NOD2 signalling pathways are critical for IL-6 production and gut homeostasis by interacting with gut microbiota [106–109]. The glucose-dependent GLP-1 release from intestinal L cells depends on the TLR4-dependent IL-6 release in male mice [110]. Thus, the involvement of TLR4 in the glucose-dependent GLP-1 release from L cells in humans must be explored for GLP-1-associated metabolic and immunological discrepancies.

Intraperitoneal MDP injection in normal chow-fed mice increases fasting GLP-1 level without affecting oral glucose tolerance [105]. However, the exact mechanism of GLP-1 production regulation in NOD2, TLR4 and CD14 KO mice subjected to inflammatory conditions, including HFD, remains to be explored. MDP-based postbiotics via NOD2 act as insulin sensitizers, as indicated by reduced adipose tissue inflammation and reduced glucose intolerance in mice with obesity without the alteration of gut microbiota and weight loss [111]. How MDP alters GLP-1 secretion in mice with HFD-induced obesity and humans with obesity needs further investigation. For example, mifamuratide (muramyl tripeptide phosphatidylethanolamine, a synthetic NOD-2 activating molecule) is an MDP-based orphan drug that induces insulin sensitization in HFD-induced obesity in mice and exploring its GLP-1 associated mechanism is critical to deal with obesity and other metabolic disorders along with its long term immunomodulatory effects [111]. Therefore, it would be interesting to observe the impact of the efficacy of GLP-1RAs in individuals with genetic mutations in their NOD2, TLR4 and CD14 genes and patients receiving NOD-2 agonists, such as mifamuratide, which is used as an orphan drug or adjuvant therapy in patients with juvenile and adolescent osteosarcoma [112].

Although hepatocytes, adipocytes and skeletal muscles do not express GLP-1R, even GLP-1 or GLP-1R agonists (GLP-1RAs) increase glucose uptake by these cells through unclear direct mechanisms, which may be due to increased blood flow, insulin secretion, neuromodulation and change in body weight throughout treatment [113,114]. However, epicardial adipose tissue (EAT) from patients with coronary artery disease (CAD) overexpresses GLP-1Rs (GLP-1R and GLP-2R) [115,116]. The EAT of patients with CAD overexpresses GLP-2R in comparison to GLP-1R expression, where GLP-2R is associated with increased fatty acid synthesis (FAS), and GLP-1R promotes fatty acid oxidation (FAO) and the transition of white adipose tissue (WAT) to brown adipose tissue (BAT) [116]. Thus, GLP-1 agonists can decrease CAD risk in patients with obesity and T2DM. However, EAT located in atrioventricular and interventricular grooves or myocardium and the visceral layer of the epicardium comprises adipocytes, nerve tissue, immune (macrophages, mast cells and CD8^+^ T cells) and stromovascular cells [117].

The EATs of CAD patients have increased numbers of pro-inflammatory M1 macrophages, mast cells and CD8^+^ T cells. In contrast, EATs of patients with obesity or T2DM have higher numbers of CD4^+^ T and B cells and pro-inflammatory cytokines, such as IL-1, IL-6, TNF-α and IFN-γ [117,118]. It is critical to note that the anatomy and transcriptome of the EAT differ from those of subcutaneous and other visceral adipose tissues (SAT and VATs) [117,119]. The lipogenesis (higher) and glucose uptake (lower) in EAT differ from those of other VATs in response to insulin, which contributes to local insulin resistance in coronary arteries due to a pro-inflammatory environment as indicated by the expression of innate immune regulators of inflammation, such as receptor for advanced glycation end product (RAGE), high mobility group box 1 protein (HMGB1), TLR4 and MyD88 (myeloid differentiation primary response 88) and reduced glucose transporter 4 (GLUT4), adiponectin and glyoxalase 1 (GLO1) [118,120,121]. Thus, it would be interesting to observe the GLP-1R expression on the immune cells of EAT for a direct GLP-1/GLP-1R axis controlling immune functions in patients receiving GLP-1RAs.

Intraepithelial lymphocytes (IELs) are present in gastrointestinal, respiratory, urinary and reproductive tract epithelium and are predominantly CD3^+^CD8^+^ T cells at most epithelial linings of the tissues but not all sites [122,123]. IELs were first described in 1884 by Weber in the small intestine epithelium as primary cells responsible for nutrient absorption [124]. These IELs can be TCRαβ and TCRγδ positive [123]. Interestingly, intestinal IELs also express self-reactive TCR. These IELs exhibit alloreactivity and cytotoxic action against invading pathogens [122]. Furthermore, subsets of IELs help for effective B-cell-mediated immune response to maintain oral immune tolerance and epithelial function. These intestinal IELs can be natural, called type-β IELs (TCRγδ^+^ IELs and CD8αα^+^TCRαβ^+^ IELs), and induced IELs called type-α IELs, which include CD4^+^TCRαβ^+^ IELs and CD8αβ^+^TCRαβ^+^ IELs [125,126]. Details of IELs in immunity and inflammation have been discussed elsewhere [123,125–127]. Interestingly, in mice, intestinal epithelium innate-like T-IELs comprise up to 80% of total IELs, but in human intestines, they comprise 5–30% of intestinal IELs and rarely express CD8αα [123]. Therefore, adult human intestinal IELs comprise mainly induced TCRαβ CD8αβ (approx. 80%) and TCRαβ CD4 (approx. 10%) T-IELs [124]. Thus, IELs are critical components of immunity at epithelial mucosal surfaces, and factors impacting them potentially deteriorate mucosal epithelial surface immunity.

**Figure 3.**
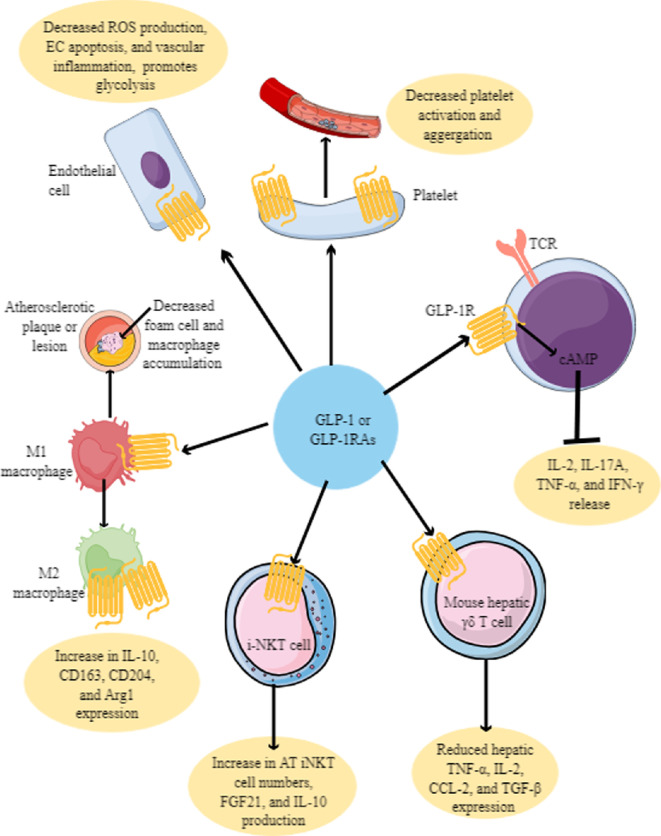
Impact of GLP-1 on different immune cells. GLP-1 induces a stage of anti-inflammation in most immune cells via direct/direct interaction with GLP-1R expression. For example, GLP-1 suppresses the release of pro-inflammatory molecules (IL-12, IL-17A, TNF-α and IFN-γ release) from IELs. In mice, GLP-1R expression in hepatic γδ T cells has been observed, and GLP-1 suppresses their pro-inflammatory function to protect from liver inflammation. However, it remains to be explored in humans. GLP-1 also induces an anti-inflammatory effect on iNKT cells in patients with obesity. Murine and human macrophages express GLP-1R. The macrophage GLP-1/GLP-1R interaction suppresses their infiltration to the site of inflammation (atherosclerotic plaque), decreases M1 macrophages and foam cell population and increases M2 macrophage number and anti-inflammatory function. GLP-1/GLP-1R interaction in ECs prevents their apoptosis and vascular inflammation, decreases ROS production and maintains their glycolysis, which is critical for survival and function. GLP-1/GLP-1R interaction in platelets suppresses their activation and aggregation, pro-inflammatory action and maintains vascular endothelial integrity by suppressing EC and platelet pro-inflammatory function. Thus, the overall GLP-1/GLP-1R axis in immune cells suppresses their pro-inflammatory function. See text for details.

Recent studies have indicated that T-cell receptor (TCR)αβ and TCRγδ-positive IELs express GLP-1R, and their treatment with exendin-4, a GLP-1RA, activates cyclic adenosine monophosphate (cAMP) pathway and suppresses the release of pro-inflammatory cytokines (IL-2, IL-17A, TNF-α and IFN-γ) (figure 3) [128,129]. The loss of GLP-1R^high^ gut IELs increases systemic GLP-1 level as exhibited by GLP-1R^−/−^ mice [129,130]. Furthermore, GLP-1R^−/−^ mice exhibit altered gut microbiota and an increased tendency to develop dextran sodium sulfate (DSS)-induced colitis due to disrupted intestinal epithelial barrier. Interestingly, the GLP-1/GLP-1R signalling axis is not critical for developing and recruiting IELs at the intestinal epithelium. In T cells, temporary and continuous increases in the cAMP levels serve as an inhibitory second messenger via cAMP/protein kinase A (PKA)/COOH-terminal Src kinase (Csk) signalling pathway and induce an anergy-like state [131–133]. Furthermore, elevated cAMP levels involve intracellular oxidation/reduction environment dysregulation in T helper 1 (Th1) cells by reducing the intracellular catalase activity and reduced glutathione (GSH) levels [134]. A recent study has identified that GLP-1RA suppresses proximal TCR signalling mediated by PKA in mouse gut IELs to suppress local and systemic T-cell-mediated inflammatory cascades, including the IFN-γ release [135].

It is critical to note that the GLP-1/GLP-1R axis on gut IEL-mediated anti-inflammatory effects is not dispensable for metabolic homoeostasis [135]. cAMP and PKA activation also inhibit TCR and CD28 co-receptor downstream signalling pathways in T cells by downregulating mitogen-activated protein kinase (MAPK), extracellular signal-related kinase (ERK) and c-Jun N-terminal kinase (c-JNK) pathways [136,137]. Therefore, identification of the GLP-1/GLP-1R axis and associated immunosuppression mechanism in human intestinal IELs will be critical to target intestinal inflammatory diseases, such as inflammatory bowel disease (IBD) or Crohn’s disease (CD) and coeliac disease, where IEL overactivation plays a significant factor in the pathogenesis [138–140]. Furthermore, γδ IELs maintain intestinal epithelial homeostasis by releasing growth factors, including keratinocyte growth factor 1 (KGF1, an epithelial cell mitogen); therefore, it becomes critical to investigate the impact of GLP-1/GLP-1R axis on IEL-mediated gut epithelium homoeostasis maintenance during normal physiology and different GI infections, which may cause sepsis through leaky gut and inflammatory diseases, such as IBD and ulcerative colitis (UC) [141–144].

Studies have indicated that the Western/HF diet (WD or HFD) reduces IELs in the small intestine within four–eight weeks in mice, and people with high body mass index (BMI) also exhibit reduced GI IELs, which is not dependent on chronic TNF-α production [145,146]. The WD reduces GI IEL density by activating farnesoid X receptor (FXR) and GI phagocytes, producing type 1 IFNs [145]. Furthermore, childhood obesity may impact GI IEL seeding and make them more prone to developing IBD, as indicated by experimental findings [146]. WD also induces hypothalamus inflammation even before the signs of obesity, such as substantial weight gain and rise in blood glucose levels due to lower GLP-1 production, as elevated GLP-1 protects from the WD-induced hypothalamus inflammation by exerting anti-inflammatory action through GLP-1Rs expressed on astrocytes of the hypothalamus [91,147]. However, gut microbiota plays a critical role in GI GLP-1 production, and obesity alters normal gut microbiota, which may lower GLP-1 production [91,148–151]. Therefore, it would be interesting to investigate the impact of GLP-1 in intestinal IEL development and homing as GLP-1 level decreases in patients with obesity and T2DM [152].

*γδ T cells*, which mainly develop in the thymus, comprise 0.5–6% of circulating T cells in humans but are higher in mucosal organs and epithelial surfaces [153,154]. γδ T cells are borderline of innate and adaptive immune cells [153,154]. In addition to the thymus, γδ T cells present in the intestinal epithelium (γδ IEL T cells) and liver develop locally in the gut cryptopatches and liver [155,156]. Vδ2γδ T cells infiltrating the liver undergo clonotypic expansion and differentiation, secrete polyfunctional cytokines, and unlike circulating γδ T cells response to TCR engagement and innate immune stimuli [157]. Liver γδ T cells comprising 3–5% of total liver lymphocytes and 15–25% of total liver T cells are critical players in liver infections and inflammatory diseases, such as non-alcoholic fatty liver disease (NAFLD), liver fibrosis, cirrhosis, liver cancer and regeneration [158–160]. Murine hepatic γδ T cells express GLP-1R and GLP-1RA (semaglutide) treatment reduces hepatic TNF-α, IL-2, CCL-2 and TGF-β expression (figure 3) along with reducing triglyceride and collagen accumulation in HFD fed mice [161]. Therefore, it would be interesting to delineate the GLP-1/GLP-1R interaction-dependent anti-inflammatory action on γδT cells, such as cAMP activity and levels. For example, prostaglandin E2 (PGE2) inhibits TCR-induced γδ T cell-mediated cytotoxic action by activating the cAMP/PKA type-1-dependent signalling pathway [162].

γδ T cells are critical producers of TNF-α, IL-2, TGF-β, IL-17 and IFN-γ along with different growth factors, such as insulin-like growth factor (IGF), platelet-derived growth factor (PDGF), vascular endothelial growth factor (VEGF) and epidermal growth factor (EGF), which play crucial roles in the inflammatory immune process [153,163]. Thus, it would be interesting to investigate the existence and impact of the GLP-1/GLP-1R axis in the functioning of human γδT cells.

Invariant natural killer T (iNKT) cells are critical innate-like T cells expressing TCRs, which recognize lipid (self and non-self) antigens presented by cell surface CD1d molecule and facilitate cytotoxic T-cell function [164,165]. GLP-1 increases iNKT cell number (figure 3) and activity in adipose tissues to promote the fibroblast growth factor 21 (FGF21) by adipocytes and promotes weight loss in mice by inducing browning of white adipose tissue (WAT) [166]. iNKT cell activation induces weight loss without affecting food intake but promotes thermogenesis and fatty acid oxidation (FAO). GLP-1 has also been shown to activate human iNKT cells, and iNKT-cell-deficient mice fail to produce FGF21 upon treatment with GLP-1RA (liraglutide) and lose less weight as compared to wild-type (WT) mice [166]. GLP-1/GLP-1RA axis activation in iNKT cells increases their anti-inflammatory function by inducing the synthesis and release of IL-10 (figure 3). NK1.1^−/−^ iNKT cells exclusively secrete IL-10 in response to free fatty acids (FFAs) via inositol-requiring enzyme 1 a (IRE1a)-X-box binding protein 1 (XBP1) arm of the unfolded protein response (UPR) in the AT to support anti-inflammatory environment [167]. Thus, iNKT cells are critical anti-inflammatory and immunoregulatory immune cells in the AT environment and protect against HFD-associated obesity and metabolic syndrome by producing IL-10 (supports M2 macrophages and regulatory T cells or T_regs_) and IL-2 (regulates helper T-cell function) [168–170]. Furthermore, KLRG1^+^ iNKT cells of the AT differentiate into CX3CR1^+^ cytotoxic cells, which specifically target and kill enlarged and inflamed adipocytes, and recruit macrophages by secreting CCL5 [171]. However, high lipid content in the AT reprogrammes anti-inflammatory iNKT cell activity to pro-inflammatory to create a pro-inflammatory AT environment to promote metabolic syndrome [172]. For example, adipose iNKT17 cells, by secreting amphiregulin (AREG), stimulate adipose stem cell proliferation and promote adipogenesis [171]. Thus, the GLP-1/GLP-1R axis has the potential to modulate iNKT cell function in different inflammatory and infectious diseases, where iNKT cells serve as potent pro-inflammatory immune cells. However, iNKT cell-dependent protective role in obesity and other metabolic syndrome-associated diseases, such as steatohepatitis, is gender-specific as CD1d^−/−^ male BALB/c mice develop more severe disease phenotype than WT male mice, which is less severe in CD1d^−/−^ female mice [173]. Another study has further indicated that FGF21 is critical for weight loss in male mice fed with a high carbohydrate diet upon treatment with GLP-1RA (liraglutide), and studies have indicated that iNKT cells are critical for the release of FGF21 from adipocytes [166,174]. Furthermore, FGF21 exerts a gender-specific effect on weight loss and hepatic lipid metabolism; for example, FGF21-treated males maintain lean mass by increasing lipid catabolism, whereas females conserve fat mass at the expense of reduced lean mass [175,176]. Hence, it would be interesting to observe the iNKT cell- and FGF21-dependent weight loss in humans taking GLP-1R agonists depending on their gender.

Myeloid immune cells (MICs, such as macrophages, DCs, neutrophils, myeloid-derived suppressor cells (MDSCs) and platelets) are critical components of innate immunity and regulators of adaptive immune response. A recent study has confirmed the expression of GLP-1R in murine macrophages isolated from C57BL/6 mice, which further increases in M2 macrophages and Ly6C^+^ macrophages [177]. GLP-1R-deficient macrophages have reduced migration properties and overexpress IL-6 without any changes in IL-1β expression, as shown in monosodium urate (MSU)-induced peritonitis in mice. GLP-1R^−/−^ mice with MSU-induced peritonitis have significantly reduced M2 macrophage infiltration/recruitment in comparison to M1 macrophages, which are the same in WT and GLP-1R^−/−^ mice with no changes in neutrophil infiltration [177]. Thus, the macrophage GLP-1/GLP-1R axis is a critical mediator of the inflammatory migration of macrophages at the site of inflammation. For example, in human macrophages, the GLP-1/GLP-1R axis induces anti-inflammatory M2 macrophage phenotype, such as induction of IL-10, CD163, CD204 and arginase 1 (Arg1) (figure 3) and downregulation of iNOS expression as indicated by signal transducer and activator 3 (STAT3) activation and overexpression [178–180]. Thus, the GLP-1/GLP-1R axis in macrophages exerts anti-inflammatory action by inducing polarization of M1 to M2 macrophages, and its dysregulation exaggerates inflammation (figure 3).

Furthermore, the GLP-1/GLP-1R axis prevents the generation of excess oxidized-low density lipoprotein laden (OxLDL) macrophages called foam cells by suppressing acyl-CoA:cholesterol acyltransferase 1 (ACAT1) expression in the atherosclerotic lesion and macrophage infiltration as well (figure 3) [181]. Human macrophages and foam cells express lower GLP-1Rs than monocytes. An *in vitro* study has indicated that liraglutide downregulates TNF-α and IL-1β gene expression in THP-1 cells (a human macrophage cell line isolated from leukemia patients) [182]. However, the authors did not see GLP-1R expression in THP-1 cells. The peripheral blood monocytes (PBMCs) isolated from patients with T2DM taking liraglutide (1.8 mg day^−1^ for 26 weeks) overexpress CCL5. However, the CCL5 increase in T2DM patients receiving liraglutide is insignificant compared to placebo group patients [182]. GLP-1R expression on human PBMCs was undetectable in this study [182]. However, macrophage function, including their polarization, depends on their tissue-/organ-specific localization; therefore, it is critical to investigate the existence and downstream signalling of the GLP-1/GLP-1R axis in diverse macrophages in different tissue/organ systems [183–185].

A flow cytometer-based study has indicated the presence of GLP-1R^+^ *neutrophils* (10%) and *eosinophils* (5%) in healthy human adults and the GLP-1R^+^ eosinophil population decreases to 2% in patients with allergic asthma [186]. Furthermore, GLP-1RA treatment to mild-asthma patient-derived eosinophils stimulated with LPS decreases IL-4, IL-8 and IL-13, but not IL-5 *in vitro*. Recently, a case report from Colombia has indicated the development of eosinophil fasciitis in a 42-year-old female patient taking weekly semaglutide injections for weight loss [187]. The condition was reversed after semaglutide discontinuation and supportive immunosuppressive agents. Several other studies have reported the development of peripheral eosinophilia, eosinophil-rich bullous pemphigus, acute interstitial nephritis, eosinophilic panniculitis and eosinophilic hepatitis in patients taking GLP-1RAs [188–193]. Most recently (14 April 2025), Pfizer has announced the discontinuation of its oral GLP-1 agonist (Danuglipron or PF−06882961) development due to severe liver injury in one patient in its phase 3 clinical trial (https://www.pfizer.com/news/press-release/press-release-detail/pfizer-provides-update-oral-glp-1-receptor-agonist). Eosinophils play a protective role in obesity, and in humans with obesity, eosinophils modulate glucose metabolism [194].

Further study has indicated that the restoration of adipose tissue eosinophils (ATEs) by adoptive transfer of eosinophils from young mice to aged obese mice dampens age-related local and systemic low-grade inflammation, a hallmark of obesity, partially through IL-4 secretion [195]. The decreased IL-4 production by eosinophils in ATs of patients with obesity have decreased eosinophil number, low IL-4 production and hyperleptinemia [196]. GLP-1RA-mediated IL-4 release from eosinophils indicated above may alter the beneficial effects of eosinophils as strategies are being developed to target obesity through eosinophils and sympathetic fat [197]. Therefore, it is critical to understand the GLP-1/GLP-1R axis in eosinophils along with other immune cells to delineate the pathogenesis of eosinophil-mediated adverse events in patients undergoing GLP-1RA-based therapies and its use during parasitic infections.

*Platelets* are critical for hemostasis, coagulation and immune homeostasis. GLP-1RAs exert antiplatelet action *in vitro* and *in vivo*, independent of GLP-1R activation by increasing NO^.^ production [198]. However, murine and human platelets express GLP-1Rs [52,198–200]. Interestingly, GLP-1R expression in murine platelets is higher than that of leukocytes [199]. Thus, other immune cells may exert the antiplatelet effect of GLP-1RAs. For example, treatment of co-cultures of platelets and monocytes (expressing GLP-1Rs) with GLP-1RA inhibits reactive oxygen species (ROS) production in monocytes and platelet activation [52]. For example, IL-10 production from cultured monocytes inhibits platelet aggregation/activation and their inflammatory function. Hence, GLP-1RA-induced inhibition of platelet aggregation and inflammatory events remains to be investigated. Increased platelet activation may induce adverse events, such as coagulation disorders and impaired immunity in patients without obesity and atherosclerosis. Further study has indicated that the blood platelets isolated from adults with obesity and American Diabetic Association (ADA) Criteria defined prediabetes show decreased aggregation and pro-inflammatory function upon stimulation with thromboxane A2 (TXA2, a pro-inflammatory mediator released by endothelial cells, macrophages and activated platelets) in the presence of GLP-1RA (liraglutide) *in vitro* and *in vivo* (figure 3) [200]. Additionally, liraglutide treatment decreases platelet activation and recruitment along with airway resistance in lysine-aspirin (Lys-ASA)-induced murine aspirin-exacerbated respiratory disease (AERD) model and in human patients with and without AERD (figure 3) [199]. However, under physiological conditions, the antiplatelet effect of native GLP-1 (7−36)) depends on the sheer flow of the blood independent of platelet GLP-1R, plasma factors and circulating leukocytes [201]. Thus, platelet GLP-1/GLP-1R interaction depends on the physiological status and the platelet location, such as circulation and the target organ. Future studies will reveal GLP-1/GLP-1R-dependent platelet functions and immune alteration during health and disease.

Endothelial cells (ECs) are considered innate immune cells depending on their different immunological characteristics and functions, such as endothelial plasticity, expression of different pattern recognition receptors (PRRs), cytokine and chemokine release, direct interaction with different immune cells and serving as antigen-presenting cells to T cells [38,202,203]. Furthermore, the endothelium is considered as an active regulator of glucose and lipid metabolism by regulating the transport and availability of insulin to different cells, such as neurons, adipocytes and myocytes [204]. Therefore, it is critical to explore and understand the GLP-1/GLP-1R axis in ECs comprising vascular endothelium.

The GLP-1 (exenatide, a GLP-1 analogue)/GLP-1R interaction in human umbilical vein ECs (HUVECs) decreases ROS generation and their apoptosis under high glucose and homocysteine-induced oxidative stress/endothelial dysfunction to exert antioxidant and anti-inflammatory effect and in coronary ECs of patients with T2DM (figure 3) [205,206]. During homocysteine-induced EC oxidative stress, exendin-4 decreases the endoplasmic reticulum (ER) stress by activating AMPK, which further increases the endoplasmic reticulum oxidoreductase (ERO1α, an essential ER chaperone in endothelial cells) expression [207].

Exenatide treatment decreases circulating adhesion molecule (sICAM-1 and sVCAM-1) levels, indicating the protective impact on coronary ECs. GLP-1 and exendin-4 treatment to HUVECs increases endothelial nitric oxide synthase (eNOS) level and NO^.^ production by increasing cytosolic cAMP level and maintaining normal vascular function [206,208]. Furthermore, exendin-4 induces AMPK and Akt phosphorylation to induce eNOS activation and NO^.^ production.

The GLP-1/GLP-1R interaction in HUVECs exerts an antioxidant effect by decreasing the NADPH oxidase activation as indicated by the reduced gp91 and human neutrophil cytochrome B light chain (CYBP or p22^phox^) expression, which are critical for NADPH oxidase activity to generate ROS [205,209]. Furthermore, GLP-1/GLP-1R interaction in HUVECs increases glucokinase (converts cytosolic glucose to glucose-6-phosphate) activity, which is a critical determinant of glucose metabolism by glycolysis (figure 3) [205,210]. Meanwhile, GLP-1R antagonist (exendin (9-39)) inhibits the increased glucokinase activity in HUVECs exposed to a high glucose environment [205]. In a murine model of arterial hypertension, liraglutide, a GLP-1RA, normalizes blood pressure, cardiac hypertrophy, vascular fibrosis, endothelial dysfunction, oxidative stress and vascular inflammation through the GLP-1/GLP-1R axis (figure 3) [211]. Furthermore, liraglutide inhibits the leukocyte–EC interaction, which decreases leukocyte and MIC migration at the inflammatory site. Interestingly, endothelial GLP-1R is critical to regulate vascular inflammation (figure 3) but not the MIC GLP-1R [211]. Further study has indicated that EC and haematopoietic lineage (HL) cell GLP-1Rs are not critical for the antiatherogenic effects of GLP-1RAs, but Tie2-targeted GLP-1R^+^ cells are critical for the anti-inflammatory action of semaglutide in the liver [161].

Interestingly, ECs, due to their low relative mitochondria numbers, depend on glycolysis for their energy demand under normal conditions, but further increase in glycolysis during inflammatory conditions support their pro-inflammatory action, which can be detrimental to the host under hypoxic, uncontrolled and irreversible inflammatory conditions [38,212,213]. Thus, GLP-1R antagonists have the potential to target ECs by targeting their metabolic reprogramming, supporting their pro-inflammatory function. However, tissue-dependent heterogeneity among vascular ECs should always be considered when targeting particular organ-specific inflammatory conditions [214]. For example, HUVECs and blood outgrowth ECs (BOECs) exhibit great heterogeneity in their proliferation and differentiation process, as indicated by the extremes of their proteomic phenotypes [215]. A recent study has indicated the higher expression of GLP-1R in normal human retinal ECs, which decreases in patients with T2DM [216]. The GLP-1RA treatment restores GLP-1R expression, improves retinal degeneration and vascular integrity in diabetic mice. The improved mitochondrial functions by GLP-1Rs in retinal ECs also inhibits pro-inflammatory STING signalling in response to cytosolic double-stranded DNA (dsDNA), which correlates well with levels of angiogenic and inflammatory molecules in retinal ECs [216]. Further findings indicate the importance of downstream cAMP response element binding protein (CREB) to the GLP-1/GLP-1R axis to suppress inflammatory STING signalling in response to mitochondrial damage. STING signalling is a critical inflammatory signalling pathway in immune cells; therefore, investigating the impact of the GLP-1/GLP-1R axis is essential.

## 5. Future perspectives and conclusion

GLP-1 or GLP-1RAs have become the drug of choice for T2DM and obesity due to their anorexigenic effects. Advances in GLP-1 and GLP-1R biology and pharmacological targeting have increased their efficacy by decreasing the GLP-1RA dose frequency for patients with T2DM and obesity. Further advances have developed single molecule co-agonists for GLP-1R and GIPR with a better efficacy against obesity and T2DM than GLP-1RA alone [217–219]. Long-acting GIPR agonists and GIPR–GLP-1R co-agonists act on the GABAergic neurons of the hypothalamus and hindbrain via GIPR signalling to induce their anorexic, anti-obesity and anti-diabetic effects in male mice [220]. Furthermore, studies have indicated that GABRA-5 positive neurons (distinctive GABAergic populations of neurons with decreased pacemaker firing in HFD-induced obesity in male mice) in the LH regulate diet-induced obesity via astrocytic GABA [221,222].

HFD in male mice induces astrocytic monoamine oxidase b (MAOb)-mediated production and release of GABA, which inhibits GABRA-5 positive neurons. However, the release of GABA from hypothalamic astrocytes triggers GABA_B_ receptors on microglia in the early postnatal brain that may induce behavioural abnormalities, which have been observed in mice lacking GABA_B_ receptors due to aberrant activation of developmental programmes [223,224]. For example, neuron–glia interaction and synaptic promiscuity are critical determinants of neural circuit formation and brain development, regulating the behavioural development of newborns [225–227]. Thus, it is interesting to explore the impact of GIPR-GLP-1R co-agonists on neurodevelopmental and behavioural aspects of newborns to women with obesity undergoing T2DM and obesity treatment and their impact on childhood/adolescent obesity and T2DM. This can be supported by the impact of GLP-1 agonists on the cognitive and mental health disorders of adults. For example, several stakeholders are advocating a better assessment of GLP-1RAs’ safety profile from the neuropsychiatric perspective, as some studies have indicated their association with cognitive impairment and increased (0.6%) suicidal events [228]. Furthermore, maternal immune alteration during pregnancy is well associated with offspring neurodevelopmental disorders (NDDs), including attention-deficit/hyperactivity disorder (ADHD), and children with NDDs also exhibit immune dysregulation, such as increased M1/M2 macrophage activity, IL-1 signalling and inflammatory response system (IRS) and compensatory immune-regulatory system (CIRS) ratio [229–231]. Therefore, the impact of GLP-1RAs on newborns (mothers taking GLP-1RAs during their pregnancies) and adults under treatment with GLP-1RAs must be followed up for any neurobehavioural and cognitive impairment.

Furthermore, the OB/GLP-1/GLP-1R axis has also been shown to regulate pancreatic insulin release in response to food intake and odour-evoked cephalic phase insulin release (CPIR) [10,232]. The activation of GABAergic neurons, which release GABA in the LH in response to OB/GLP-1/GLP-1R axis activation, inhibits sympathetic nerve activation in the pancreas for releasing insulin. However, the impact of GABA on local microglia in terms of immunological functions is divided into some anti-inflammatory (inhibition of pro-inflammatory cytokine (TNF-α, IL-6 and IL-12p40) release) and pro-inflammatory (NLRP3 and NF-κB activation) findings [233–235].

Furthermore, FXRs, which are activated by bile acids (BAs), are also expressed by IEECs or L cells [236,237]. FXR activation in L cells decreases proglucagon content and hence the GLP-1 production by intervening with the glucose-responsive factor carbohydrate-responsive element binding protein (ChREBP) and inhibiting glycolysis. BAs, such as lithocholic acid via G-protein-coupled bile acid receptor 1 (GPBAR1), increase L cell differentiation and elevate GLP-1 secretion [238]. However, a synthetic GBPAR1 agonist needs intact GLP-1R and serotonin-5-hydroxytryptamine receptor 4 (5-HT-4) signalling. Interestingly, the serotonin signalling by the 5HT-4 receptor mimics the effect of GBPAR1, working downstream of GLP-1. Hence, people with BA imbalance or overproduction, such as patients with cholestasis, BA malabsorption in the intestine (colon), chronic pancreatitis, celiac disease (CD), small intestinal bacterial overgrowth (SIBO), depression and memory-associated disorders, may have altered GLP-1 production [239–242]. Furthermore, FXR knockout (KO) mice lose the efficacy of TLR9 agonists against 2,4,6-trinitrobenzene sulfonic acid (TNBS)-induced colitis. In contrast, FXR activation in TLR9 and MyD88 KO mice rescues mice colitis by decreasing inflammation in response to interferon regulatory factor-7 (IRF7) recruitment at the FXR promoter site [243]. Thus, it would be interesting to observe the specific FXR activation in intestinal L cells, GLP-1 release and impact on local immune cells such as IELs, including γδT cells. Furthermore, how intestinal GLP-1 alteration and exogenous GLP-1RAs (approved for obesity and T2DM patients) affect the impact of BAs and microbiota interaction-dependent shaping of the host immunity and vice versa should be investigated as these patients have altered gut microbiota [244–246].

Furthermore, GLP-1 basal level does not vary between sexes, but females show higher GLP-1 levels than males following an oral glucose tolerance test (OGTT) [247], and female GLP-1 level increases immediately after moderate-intensity continuous exercise (MICT) and sprint interval training (SIT), which is absent in males (heavier, taller and leaner than females) with similar BMI [248]. Females with obesity taking GLP-1RAs show more prominent weight loss (have more prominent gastrointestinal adverse events) than males with obesity [249]. The gut microbiota also varies between sexes; for example, the gut microbiota of premenopausal women is highly diverse, with a higher abundance of multiple species known to have beneficial effects on host metabolism than men of the same population (China, Israel and The Netherlands), linking sex hormones, gut microbiota and host metabolism, affecting immunity [250–252]. Thus, the GLP-1/GLP-1R axis may serve as a missing link for gender, gut, brain, microbiota, metabolism and immunity as GLP-1RAs have the potential to modulate the immune response directly through immune cells expressing GLP-1Rs and via altering metabolism and neurotransmitter release. For example, human studies have indicated that acute intravenous GLP-1 administration does not affect reproductive hormone (luteinizing hormone (LH) and testosterone) secretion in healthy men during an euglycaemic clamp but decreases testosterone secretion pulse duration [253,254]. However, men with obesity, T2DM and hypogonadism receiving GLP-1RAs have shown increased circulating testosterone levels, improvement in erectile dysfunction and conventional sperm parameters [255–257].

Therefore, it is imperative that we conduct further studies to avoid gender and immune-based adverse events in patients taking GLP-1RAs. Understanding the immunomodulatory actions of the GLP-1/GLP-1R axis targeted by GLP-1RAs is crucial for ensuring the safety and efficacy of these treatments.

## Data Availability

This article has no additional data.
